# Association between Sick Days and Psychological Problems Among Teachers in Canada

**DOI:** 10.1192/j.eurpsy.2025.1803

**Published:** 2025-08-26

**Authors:** A. Belinda, Y. O. Wei, R. D. L. D. Dias, A. Orimalade, P. B.-M. Brett-MacLean, V. I. O. Agyapong

**Affiliations:** 1 University of Alberta, Edmonton; 2 Dalhousie University, Halifax, Canada

## Abstract

**Introduction:**

Increased sick leave among educators can detrimentally impact students’ productivity, and academic achievement. It remains unknown whether the number of sick days taken by educators in the preceding school year correlates with the prevalence or severity of psychological problems among educators in the subsequent school year.

**Objectives:**

This study aimed to examine the number of self-reported sick days taken by educators in three Canadian provinces during the 2021/2022 academic year and its association with measures of stress, burnout, low resilience, depression, and anxiety during the 2022/2023 school year.

**Methods:**

Data was collected from educators in three Canadian provinces, Alberta, Nova Scotia, and Newfoundland and Labrador, from September 1, 2022, to August 30, 2023. The Maslach Burnout Inventory-Educator Survey (MBI-ES), the Brief Resilience Scale (BRS), and the Perceived Stress Scale were used to assess burnout, resilience, and stress, respectively. Likely Generalized Anxiety Disorder (GAD) and likely Major Depressive Disorder (MDD) were assessed using the Generalized Anxiety Disorder-7 and Patient Health Questionnaire-9 scales, respectively. Statistical analysis was conducted using SPSS version 28.

**Results:**

763 subscribers completed all the demographic, professional questions, and clinical scales, giving a response rate of 39.91%. Of these, there were 94 (12.3%) males and 669 (87.7%) females. Educators who reported taking 11 or more sick days in the previous academic year were at least three times more likely to exhibit high stress, emotional exhaustion, likely GAD, low resilience, and likely MDD than educators with no sick days during the preceding year. Similarly, educators with 11 or more sick days had significantly higher mean scores on the GAD-7 scale, the PHQ-9 scale, the PSS-10, the MBI Emotional Exhaustion subscale, and the MBI Depersonalization subscale than those with zero sick days.

**Conclusions:**

This study demonstrates a significant association between sick days and the prevalence and severity of high stress, low resilience, burnout, anxiety, and depression among educators. Short-term sick leave can escalate into long-term absences without adequate support for teachers. Governments and policymakers in the education sector must foster a supportive environment that enables teachers to thrive and effectively perform their professional role without taking prolonged sick days, which can undermine student learning and achievement.

**Disclosure of Interest:**

None Declared

